# Macroecology of Dung Beetles in Italy

**DOI:** 10.3390/insects15010039

**Published:** 2024-01-07

**Authors:** Simone Fattorini, Alessia Vitozzi, Letizia Di Biase, Davide Bergamaschi

**Affiliations:** 1 Department of Life, Health and Environmental Sciences, University of L’Aquila, Via Vetoio, 67100 L’Aquila, Italy; letizia.dibiase@graduate.univaq.it; 2 Department of Statistical Sciences, Sapienza University of Rome, Piazzale Aldo Moro 5, 00185 Rome, Italy; vitozzi.1920984@studenti.uniroma1.it; 3 Department of Entomology, Forbes 410, The University of Arizona, Tucson, AZ 85721, USA

**Keywords:** Scarabaeoidea, Scarabaeidae, Aphodiinae, Geotrupidae, beta diversity, biogeography, glacial refugia, mediterranean, peninsula effect, Pleistocene

## Abstract

**Simple Summary:**

Dung beetle communities include three groups of insects: the true dung beetles (scarabaeines), the small dung beetles (aphodiines), and the earth-boring dung beetles (geotrupids). The Italian dung beetle fauna is one of the richest in Europe due to the position of the Italian peninsula in the middle of the Mediterranean global hotspot of biodiversity. Dung beetle faunas in the Italian peninsula appear to be richer than that of Sardinia, which has a distinctly impoverished fauna due to its strong isolation from the mainland. Dung beetle species richness varies along the Italian peninsula in response to climatic factors. Aphodiines (that need to use the dung before it dries) are mainly associated with humid and cold climates and do not show a latitudinal pattern, while scarabaeines (which can cope with dry conditions) increase their diversity southward. Dung beetle species composition in Italian regions reflects both random processes of dispersal (possibly favored by human influences due to millennia of grazing activities) and the role of southern areas as refugia during Pleistocene glacials.

**Abstract:**

The Italian fauna includes about 170 species/subspecies of dung beetles, being one of the richest in Europe. We used data on dung beetle distribution in the Italian regions to investigate some macroecological patterns. Specifically, we tested if species richness decreased southward (peninsula effect) or northward (latitudinal gradient). We also considered the effects of area (i.e., the species–area relationship), topographic complexity, and climate in explaining dung beetle richness. Finally, we used multivariate techniques to identify biotic relationships between regions. We found no support for the peninsula effect, whereas scarabaeines followed a latitudinal gradient, thus supporting a possible role of southern areas as Pleistocene refuges for this group of mainly thermophilic beetles. By contrast, aphodiines were more associated with cold and humid climates and do not show a distinct latitudinal pattern. In general, species richness was influenced by area, with the Sardinian fauna being however strongly impoverished because of its isolation. Faunal patterns for mainland regions reflect the influence of current ecological settings and historical factors (Pleistocene glaciations) in determining species distributions.

## 1. Introduction

Dung beetles are a taxonomically composite group of coprophagous Scarabaeoidea belonging to Geotrupidae (known as earth-boring dung beetles or dor beetles), Scarabaeidae Scarabaeinae (known as true dung beetles), and Scarabaeidae Aphodiinae (known as small dung beetles) [1,2]; the latter two groups are sometimes regarded as deserving the taxonomic rank of family (Scarabaeidae and Aphodiidae) [3,4].

The feeding habits of all of these three taxonomic groups are extremely variable, although dung is the most commonly used food for most species [1,2,5,6,7,8,9,10]: Geotrupidae (about 400 known species worldwide) include mycophagous, phytophagous, necrophagous, saprophagous, and coprophagous species; Aphodiinae (about 3200 known species worldwide) include saprophagous, necrophagous, and mycophagous species, but most species are coprophagous; Scarabaeinae (more than 5000 known species worldwide) are virtually invariably coprophagous, although a few species are known to be saprophagous, necrophagous, or mycophagous.

Because of their peculiar trophic niche and behaviors, dung beetles are among the most iconic insects [11,12,13,14], with members of the Scarabaeinae (namely, the scarab beetle *Scarabaeus sacer* Linnaeus, 1758, and allied taxa) being universally known for their complex parental cares (which were among the first to be investigated by early entomologists interested in insect ethology [11,15,16]) and the importance that they had in the culture of Ancient Egypt [13,17,18]. Based on their nesting behavior, dung beetles are frequently grouped into three main categories: dwellers (species that feed in the dung pat as adults and lay eggs within or under the dung mass where they undergo larval development; most aphodiines belong to this category), tunnellers (species that dig chambers more or less directly underneath the pat for breeding; geotrupids and many scarabaeines), and rollers (species that form a ball of dung that can be rolled away from the pat and buried for breeding; this category is found only among the scarabaeines) [1,2,19,20,21].

By removing substantial amounts of dung, dung beetles provide essential ecosystem services, including nutritional cycling, maintenance of soil characteristics, seed dispersal, reduction in greenhouse gases, and livestock protection from dung breeding dipterans and gastrointestinal parasites [22,23,24,25,26,27,28,29,30,31,32,33,34]. Because of their sensitivity to several forms of anthropogenic impacts on their ecosystems, dung beetles are globally declining [35,36,37,38,39,40].

The Italian fauna of dung beetles includes about 19 species of Geotrupidae and 170 species of Scarabaeidae (46 Scarabaeinae, about 90 dung-feeding Aphodiinae, and about 36 Aphodiinae feeding prevalently or exclusively on resources different from dung), being one of the richest in Europe [10]. This exceptional diversity parallels that recorded for the most disparate taxa in Italy, e.g., [41,42,43,44], which represents an important hub of biodiversity within the Mediterranean global hotspot [45,46,47,48,49] because of its environmental heterogeneity and complex biogeographical history [50,51,52,53,54,55,56,57,58,59,60,61]. Thanks to a long history of entomological research, the Italian fauna of dung beetles is taxonomically and ecologically well known, and accurate distributional information are available at a coarse (regional) scale [10]. However, despite the availability of relatively good ecological and distributional data, no comprehensive study addresses the macroecology of dung beetles in Italy.

Here, we used data on dung beetle distribution in the Italian regions to test the following macroecological hypotheses:

(1) Species richness increases with area. One of the most universal biogeographical patterns is the species–area relationship (SAR), that is, the increase in species richness with increasing area [62]. Thus, we tested if dung beetle richness in Italy increased with the area of the Italian regions.
(2) Islands have depauperate faunas. The Italian territory is composed of a long peninsula and two main islands (Sicily and Sardinia). Island biotas are known to be impoverished when compared to those of mainland regions of equal size [63,64]. Thus, we tested if island dung beetle faunas were impoverished in comparison with the mainland fauna.
(3) Dung beetles conform to the peninsula effect. The Italian peninsula stretches from the European mainland at the north toward the center of the Mediterranean basin at the south. If the biota of a peninsula results from colonization processes starting from the mainland, species richness should decrease from the base to the tip of the peninsula, a phenomenon recorded for a variety of taxa and peninsulas worldwide [60,61,65,66]. In the case of the Italian peninsula, given its north–south alignment, species richness should decrease from the north (base of the peninsula) to the south (tip of the peninsula) [56,60,61]. To test this hypothesis, we correlated dung beetle richness with the latitude of the regions.
(4) Dung beetles conform to the latitudinal gradient. One of the most widespread macroecological patterns is the latitudinal gradient: species richness tends to decrease from the equator to the poles [53,55,61,67,68,69,70,71,72,73,74,75,76,77,78,79,80,81,82]. In the case of the Italian territory, this should translate to a pattern of decreasing richness from south to north, thus leading to a pattern opposite to that predicted by the peninsula effect but which might be explained by the current climate (if species respond positively to increasing temperatures) and the role of southern Italian regions as a Pleistocene refugial center. During the Pleistocene glacials, most European areas northern to the Alps were covered by ice, forcing species to retreat to the southern, ice-free areas; these areas acted as both refugial and speciation centers, from which species recolonized the northern areas after deglaciation [56,60,61]. Within the Italian peninsula, if dung beetles are positively influenced by temperatures and/or southern regions acted as Pleistocene refuges, a negative relationship between latitude and richness is expected [56].
(5) Climate influences dung beetle richness, with differences between aphodiines and scarabaeines that reflect their main ecological characteristics. To test this hypothesis, we investigated the influence of climatic characteristics of Italian regions on their dung beetle diversity. Precipitation and temperature are expected to influence dung beetle diversity because of their effects on excrement persistence and quality. In cold and humid climates, excrement degradation is slow, while it proceeds faster in hot and arid climates, thus favoring species that can protect the offspring through subterranean pedotrophic nests [1,83,84,85]. Thus, dung beetle communities of temperate areas tend to be dominated by aphodiines (which are mainly dwellers), whereas scarabaeines (which are rollers and tunnellers) predominate in tropical and Mediterranean communities [85,86]. We expect that the amount of precipitation should influence positively the aphodiine richness in Italy and the overall dung beetle richness, given the prevalence of aphodiines in the Italian fauna, but not scarabaeines. We also expect that aridity should affect negatively aphodiines and the total dung beetle richness but not scarabaeines. In general, we expect that, given their different ways of food utilization, aphodiine will be negatively influenced by temperatures, whereas scarabaeines should be positively influenced or not influenced. These contrasting responses should lead to a lack of relationship between total dung beetle richness and temperature. We also expect that climatic variability should have a negative influence on aphodiines and possibly on dung beetles in general, but not on scarabaeines, which should be more adapted to tolerate high temporal variation in temperatures.
(6) Elevation influences dung beetle richness negatively. Dung beetle diversity tends to decline with increasing elevation [1,85,87,88,89,90,91,92,93,94,95,96,97], although mid-elevation peaks [89,91,98] or a lack of relationship have been also reported [85,99]. Not only at higher elevations temperatures might be too low, especially in winter, but the drying effects of increased windiness and insulation might lead to the rapid desiccation of excrements, especially in the summer, when precipitation may be completely absent [85]. Thus, we hypothesize that more mountainous regions should have fewer species of dung beetles than the less mountainous ones, leading to an inverse relationship between dung beetle richness and regional average elevation.
(7) Species distributions may result from three, mutually non-exclusive processes: influences of current (present day) ecological conditions, random processes, and historical (paleogeographical and paleoecological) events. As among current factors, climate and topography are major drivers of species distributions, we expect that inter-regional dissimilarities in species composition (β-diversity) correlate with similarity in these environmental conditions [53,56,60,61,100]. If species distributions result from random processes (from stochastic population dynamics and spatially constrained dispersal), spatial patterns of species similarity among regions should simply reflect their geographical proximity [53,56,60,61,101]. This means that we expect a distance decay of similarity independently from ecological differences among regions. Finally, if current species distributions have been influenced by historical factors (such as the effects of glacials), this would produce regional groupings characterized by distinct biogeographical discontinuities [53,56,60,61].

## 2. Materials and Methods

### 2.1. Data Collection

**Figure 1:**
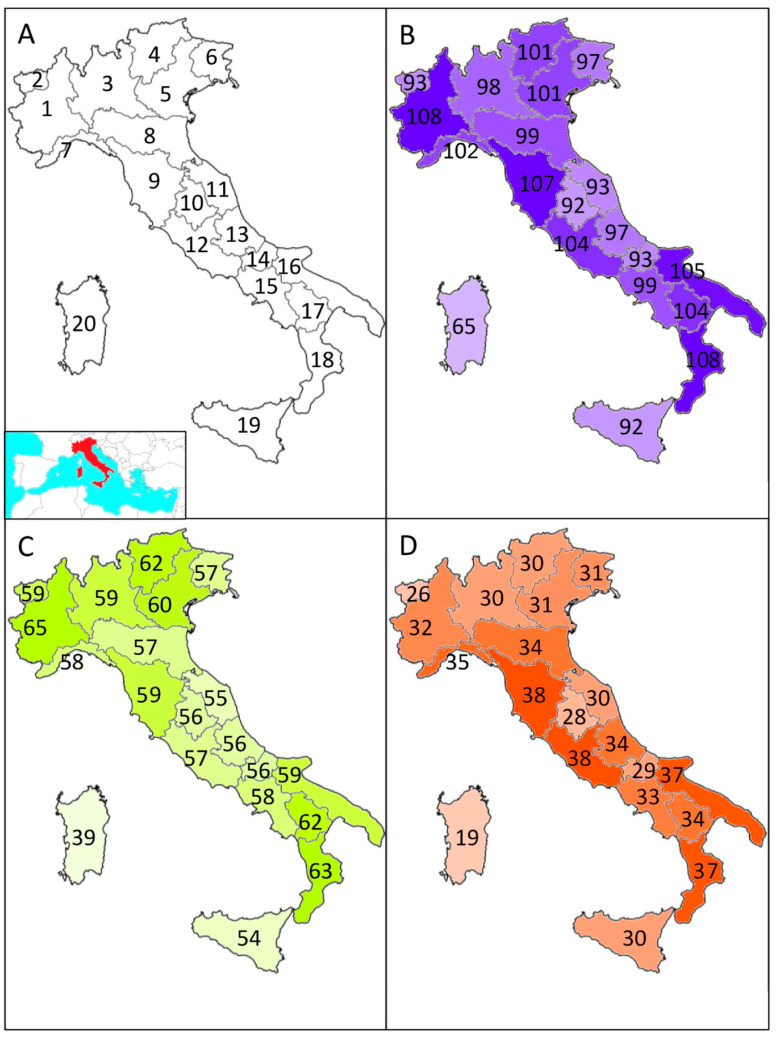
Study area (with regions identified by numbers as follows: 1: Piedmont, 2: Aosta Valley, 3: Lombardy, 4: Trentino-Alto Adige, 5: Veneto, 6: Friuli-Venezia Giulia, 7: Liguria, 8: Emilia-Romagna, 9: Tuscany, 10: Umbria, 11: Marche, 12: Latium, 13: Abruzzo, 14: Molise, 15: Campania, 16: Apulia, 17: Basilicata, 18: Calabria, 19: Sicily, 20: Sardinia) (**A**) and total number of species of dung beetles (**B**), number of species of aphodiines (**C**), and number of species of scarabaeines (**D**) in each region. The inset in panel (**A**) shows the location of Italy within the Mediterranean basin.

Dung beetle species distributions in Italy were coded as presence/absence at the regional level using information reported in Ballerio et al. [10], updated with subsequent findings retrieved from Forum Entomologi Italiani [102] (Figure 1).

Uncertain records were excluded. We only considered species that feed at least regularly on dung. We considered both species and subspecies not only because subspecies are biogeographically informative but also because there is evidence that at least some subspecies might deserve the taxonomic rank of species and the dividing line between species and subspecies in these insects is rather arbitrary [10]. The word species will be applied to both species and subspecies for simplicity. Although the use of regions as geographical units instead of regular grids is afflicted by problems arising from their different size and internal high heterogeneity in environmental conditions, regional data are well suited for biogeographical and macroecological studies [103,104,105] because they are more accurate and comprehensive than point records [106,107,108], being also robust to the violation of constant grain size [109].

Areas of the regions, their limits, and centroids (latitude and longitude, decimal degrees) were extracted from ISTAT [110]. To investigate the influence of climatic factors on dung beetle diversity, for each region, we calculated the following parameters: average of the annual mean temperatures (MEANTEMP), average of the maximum temperatures of the warmest month (MAXTEMP), average of the minimum temperatures of the coldest month (MINTEMP), average of temperature annual ranges (TEMPRAN, calculated as the average of the differences between MAXTEMP and MINTEMP values), spatial range of the mean annual temperatures (SRTEMP, i.e., the range of TEMP), average of annual precipitations (PREC), spatial range of precipitation (SRPREC, i.e., the range of PREC), precipitation of the driest quarter (PRECDQ), and spatial range of precipitation of driest quarter (SRPRECDQ). Temperatures were expressed in °C and precipitations in mm. Climatic variables were obtained from WorldClim 2.1 (period 1970–2000, resolution 30 s) [111]. Data were extracted by using ArcGis Pro 3.1.3 [112].

As topographical variables, we considered minimum elevation (MINELE), maximum elevation (MAXELE), mean elevation (MEANELE), and spatial range of elevation (SRELE, calculated as the difference between maximum and minimum values). All elevations were in m above sea level. Topographical variables were obtained from TINITALY (resolution 10 m) [113]. Data were extracted by using ArcGis Pro 3.1.3 [112].

### 2.2. Data Analysis

Analyses were conducted separately for the entire dataset (Geotrupidae, Scarabaeinae, and Aphodiinae) and for Scarabaeinae and Aphodiinae separately (Geotrupidae were not analyzed separately because of the small number of species).

The species–area relationship (SAR; hypothesis 1) was modeled using the power function, as this model describes adequately most empirical data [114,115,116].

The model is expressed by the following equation:

*S = cA^z^,* (1)

where *S* is the species number, *A* is area, and *c* and *z* are fitting parameters.

This model was applied here by using its linearized form:

ln(*S*) = ln(*c*) + *z* ln (*A*), (2)

where *c* represents the expected number of species per unit area, and *z* is the slope of the function [116].

For ease of interpretation, areas were expressed as 10^3^ km^2^. Data were fitted using ordinary least squares (OLS) regressions, both including (overall models) and excluding island regions (Sicily and Sardinia) (mainland models). Residuals of Sicily and Sardinia from the regression line of the overall model were inspected to determine whether islands had impoverished faunas, with negative residuals indicating impoverishment (hypothesis 2) [63]. Analysis of covariance (ANCOVA) was applied to aphodiine and scarabaeine mainland regressions to test for homogeneity of slopes and differences between intercepts [116].

To test the peninsula effect (hypothesis 3) and the latitudinal gradient (hypothesis 4), correlation analyses were performed between the latitude (centroid) of mainland regions and dung beetle diversity. As measures of dung beetle diversity, we used the residuals (calculated from Equation (1) after back-transformation of Equation (2)) from the mainland SAR model; the use of residuals allowed us to consider the possible influence of differences in area size among regions in determining differences in species richness.

To test the importance of climatic and topographical variables as predictors of dung beetle diversity (hypotheses 5 and 6), we adopted a multimodel inference procedure. With this approach, we constructed models representing every possible combination of explanatory variables. Then, models were ranked in increasing order of their corrected Akaike information criterion value (AICc); models with a ΔAIC ≤ 2 were considered as equally supported and averaged using both full and conditional averages. In the full average, regression coefficients for variables that are not included in a given model are set to zero, whereas conditional average only averages over the models where the parameter appears. Analyses were conducted using residuals of richness from Equation (1) as SAR-corrected estimates of species richness. Before analyses, we checked multicollinearity among covariates using the Spearman correlation coefficient to consider both linear and non-linear monotonic relationships, and in the case of strong collinearity (*r*_s_ > |0.6|) [117], we decided which variable to retain in the analyses as follows. For temperatures, we found pairwise collinearity between MEANTEMP, MAXTEMP, MINTEMP, and SRTEMP. Therefore, we decided to retain MEANTEMP. For precipitation, we found that PREC was strongly correlated with SRPREC, thus we retained PREC. For aridity, PREDQ was strongly correlated with SRPREDQ, and we retained PREDQ. For topographical variables, MEANELE was strongly correlated with MINELE, MAXELE, and SRELE. Thus, only MEANELE was maintained in the analyses. 

Faunal relationships between regions (hypothesis 7) were investigated using two coefficients of dissimilarity (β-diversity): the Dice–Sørensen coefficient (βsor, which expresses the total β-diversity) and the Simpson coefficient (βsim, which expresses the pure turnover component, i.e., the compositional differences after removing the effect of nestedness, that is, the compositional change caused by ordered species loss) [118,119,120,121,122,123]). Biogeographical distances were correlated with (1) environmental distances between regions calculated as Euclidean distances for the environmental variables (MEANTEMP, MAXTEMP, MINTEMP, TEMPRAN, SRTEMP, PREC, SRPREC, PRECDQ, MINELE, MAXELE, MEANELE, and SRELE) after standardization, and (2) geographical distances between regions calculated as distances (in km) between centroids of latitude and longitude. Correlations between matrices were performed using Mantel tests (to test the influence of either geographical position or environmental conditions on biogeographical distances) and partial Mantel tests (to evaluate the effect of geography, controlling for the effect of environmental conditions, and to evaluate the effect of environmental conditions, controlling for the effect of geography) [124].

To depict inter-regional biogeographical relationships expressed by Dice–Sørensen and Simpson coefficients, we used both cluster analyses and non-metric multidimensional scaling (NMDS). For cluster analyses, we used the UPGMA (unweighted pair group method with arithmetic average) as an amalgamation rule, as it is considered the clustering strategy that minimizes the distortion of the original data matrix [125], and it is therefore favored in biogeographical research (e.g., [125,126,127,128,129,130,131]). Since the resulting dendrograms and bootstrap supports are affected by the order of the regions in the original matrix (especially when pairwise distance values are equal) [132,133], we adopted a procedure which re-samples the order in which areas are introduced in the analyses and creates consensus trees in bootstrap analysis [133]. The impact of alternative topologies was evaluated by comparing the initial tree with consensus trees obtained from 1000 trees produced after the re-ordering of regions for six different consensus rules (from 0.5 to 1 with a step of 0.1), and evaluating the percentage of times that each node was repeated among different consensus rules (node strength; Figure S1). Then, a bootstrap analysis was applied to the 0.5 consensus tree, using a multiscale bootstrap procedure with selection of the scale at which strongly and weakly supported nodes are best recognized [57]. The NMDS is an ordination technique particularly suitable to disclose multiple biogeographical relationships [60,61,134,135]. Procrustes distances were used to compare solutions until a minimum stress value was obtained. For the two-dimensional representation, the axis with the highest variance was standardized between 0 and 1, and the other axis was rescaled according to the first one. Finally, the colors blue, green, yellow, and red were assigned to the four corners, and each region received an RGB (red, green, or blue) color based on its position in the two-dimensional space.

All analyses were performed in R 4.1.3 software [136] using the following packages: MuMIn 1.46.0 [137] (for multimodel inference analyses), vegan 2.6–2 [138] (for Mantel tests and NMDS), and recluster 2.9 [139] (for cluster analysis and NMDS).

## 3. Results

**Figure 2:**
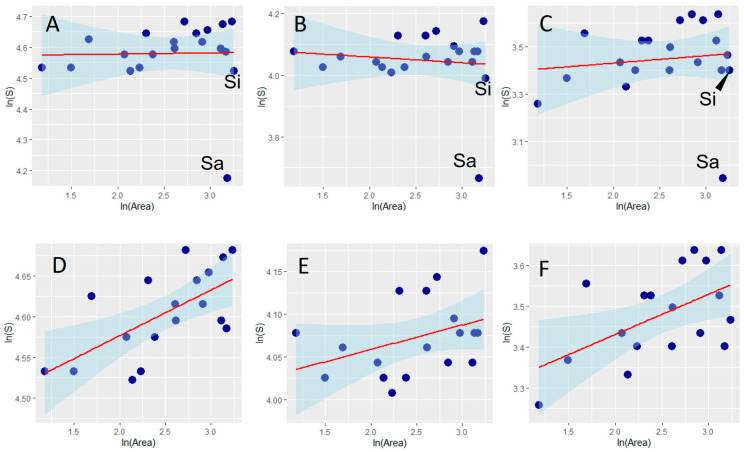
Relationships (OLS regressions) between dung beetle species richness (S) and area of Italian regions for the total fauna (**A**,**D**), and for the aphodiines (**B**,**E**) and the scarabaeines (**C**,**F**), separately. Panels (**A**–**C**) show results obtained including the islands of Sicily (Si) and Sardinia (Sa); panels (**D**–**F**) show results obtained excluding islands. Areas are measured as 10^3^ km. Both species richness and area were ln-transformed.

The species–area relationships (SARs) modeled for the total dung beetle faunas and for the aphodiines and scarabaeines analyzed separately showed that Sardinia has a strongly impoverished fauna, whereas Sicily does not seem to have substantially fewer species than expected (Figure 2, Table 1); thus, hypothesis 2 is supported for Sardinia but not for Sicily. SARs ameliorated substantially if islands are removed from the analyses, thus showing that after the confounding effect of isolation is removed (Figure 2, Table 1), species richness increases with area, as expected according to hypothesis 1. Regressions for the mainland regions have similar slopes but different intercepts (ANCOVA: homogeneity of slopes: *F* = 2.774, *p* = 0.106; differences between intercepts: *F* = 554.300, *p* < 0.0001), with aphodiines having much more species per unit area than scarabaeines (55 species for a unit area of 10^3^ km^2^ vs. 25 species for a unit area of 10^3^ km^2^).

**Figure 3:**
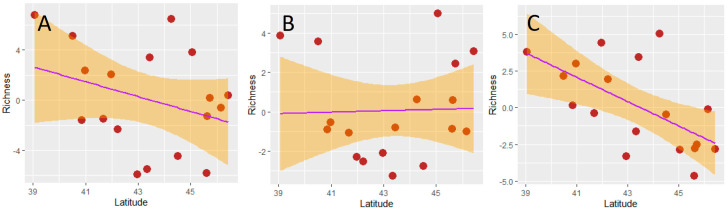
Relationships (OLS regressions) between dung beetle richness and latitude (centroid) of Italian regions: (**A**) total dung beetle richness; (**B**) aphodiine richness; (**C**) scarabaeine richness. Richness values are, in all cases, the residuals from the respective species–area relationships.

The residuals of the SARs (used as area-corrected estimates of richness) were not significantly correlated with latitude for the total dung beetle fauna and for the aphodiines, whereas a negative correlation emerged for the scarabaeines (Figure 3, Table 2). Therefore, neither hypothesis 3 nor hypothesis 4 were supported for aphodiine and total dung beetle richness, but hypothesis 3 was falsified and hypothesis 4 supported for the scarabaeines.

Latitude was correlated (Spearman correlation coefficient) negatively with annual mean temperature (*r_s_* = −0.880, *p* < 0.0001) and positively with temperature annual range (marginally, *r_s_* = −0.443, *p* = 0.067), annual precipitation (*r_s_* = −0.612, *p* = 0.008), and precipitation of the driest quarter (*r_s_* = 0.744, *p* < 0.001), but not with average elevation (*r_s_* = 0.362, *p* < 0.140).

Total precipitation influenced positively both aphodiine richness and the total richness of dung beetles, as expected according to hypothesis 5 (Table 3). By contrast, precipitation was not important for scarabaeine richness, as expected according to hypothesis 5 (Table 3).

Aridity influenced negatively aphodiine richness and total dung beetle richness, but not scarabaeine richness (thus supporting hypothesis 5) (Table 3). Temperature was not important for the total dung beetle richness and scarabaeine richness but exerted a negative effect on aphodiines (as postulated by hypothesis 5) (Table 3). Climatic instability had a negative effect on both aphodiine and total dung beetle richness (as expected by hypothesis 5) but also on scarabaeine richness (which contrasts with our assumptions) (Table 3). Elevation influenced positively aphodiine richness (which is in contrast with hypothesis 6) (Table 3).

Mantel tests (Table 4) indicated that biogeographical dissimilarities (expressed by either the Dice–Sørensen or the Simpson indices) were correlated with geographical position even after controlling for environmental distances, whereas correlation between biogeographical dissimilarities and environmental distances were weak and disappeared after controlling for geographical position. This highlights the influence of geographical distances independently from environmental characteristics (climate and topography) (thus supporting hypothesis 7 for the influence of random processes more than that of current ecological conditions).

**Figure 4:**
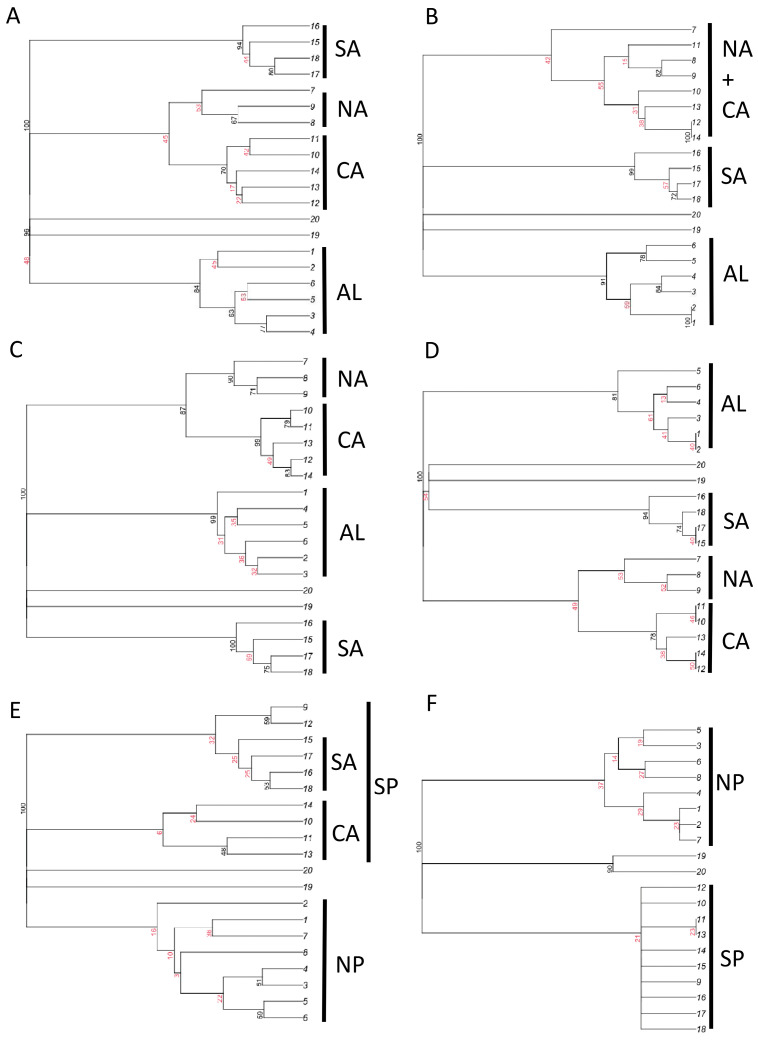
Results of cluster analyses (consensus trees) for total dung beetle species composition (**A**,**B**), aphodiines (**C**,**D**), and scarabaeines (**E**,**F**), using Dice–Sørensen (**A**,**C**,**E**) and Simpson (**B**,**D**,**F**) indices of similarity, for the Italian regions. Highly (black) and weakly (red) supported nodes are identified using multiscale bootstrap method (most nodes have a strength of 100, thus indicating unambiguous relationships; see Figure S1). AL: Alpine regions, NA: north Apennine regions, CA: central Apennine regions, SA: southern Apennine regions, NP: regions north of the Po River, SP: regions south of the Po River.

In all cluster analyses (Figure 4 and Figure S1), the two islands (Sardinia and Sicily) were distinctly separated from mainland regions. Analyses conducted using all dung beetle species with the Dice–Sørensen index showed a distinct separation of mainland regions into four groups: Alpine, northern and central Apennine, central Apennine, and southern Apennine regions (Figure 4A), whereas use of the Simpson index showed three groups: Alpine, northern and central Apennine, and southern Apennine regions (Figure 4B). When the analyses were restricted to the aphodiines, the four groups of regions were recovered using both the Dice–Sørensen and Simpson indices (Figure 4C,D). For the scarabaeines, the Simpson index indicated a clear distinction between two groups of regions: those to the north of the Po River and those to the south (Figure 4E,F).

**Figure 5:**
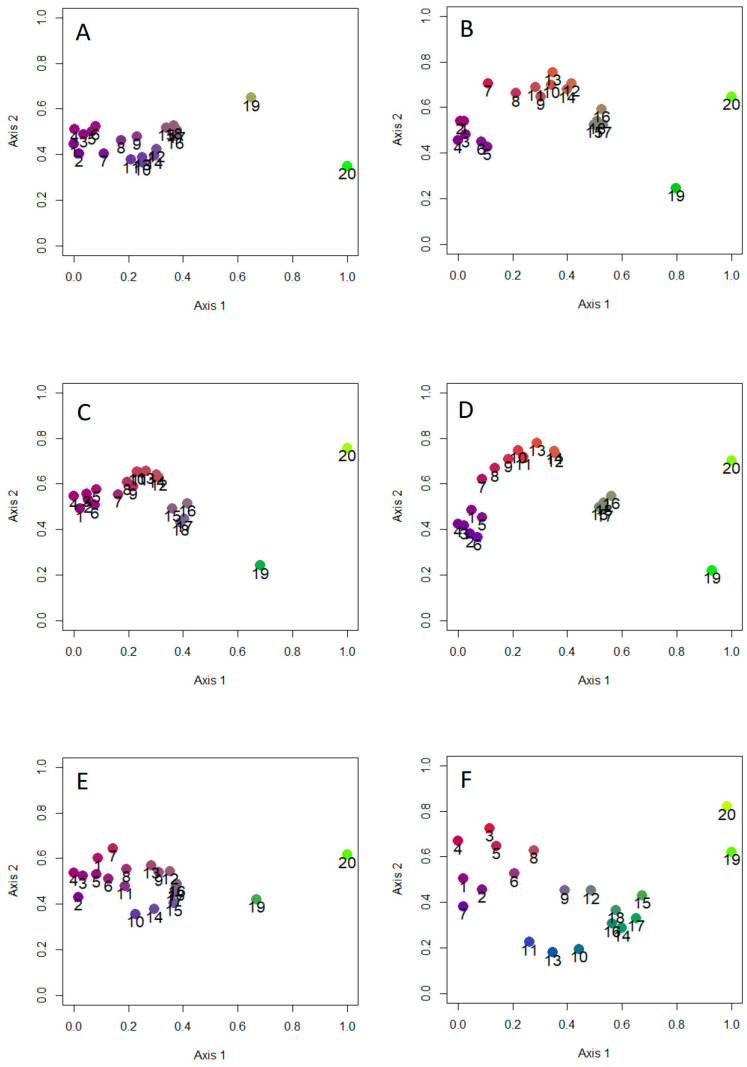
Results of non-metric multidimensional scaling for total dung beetle species composition (**A**,**B**), aphodiines (**C**,**D**), and scarabaeines (**E**,**F**), using Dice–Sørensen (**A**,**C**,**E**) and Simpson (**B**,**D**,**F**) indices of similarity, for the Italian regions. Stress values are as follows: (**A**) 0.043, (**B**) 0.078, (**C**) 0.028, (**D**) 0.049, (**E**) 0.083, and (**F**) 0.138. Regions are numbered as in Figure 1A.

The results of NMDS (Figure 5) indicated in all cases a strong separation of islands (regions 19 and 20) from the mainland. For aphodiines, NMDS results showed the presence of three mainland groups: (1) Alpine regions (regions 1–6); (2) northern and central Apennine regions (regions 7–14); (3) southern Apennine regions (regions 15–18). The northern and central Apennine regions appeared strictly associated with the Alpine ones, while the southern regions were distinctly separated, especially when the Simpson index was used. Similar patterns were found using the total dung beetle fauna. NMDS results for scarabaeines indicated the presence of two distinct groups of mainland regions corresponding to the areas north (regions 1–8) and south (regions 9–18) of the Po River. Overall, these results support the influence of historical factors that created biogeographical discontinuities (hypothesis 7).

## 4. Discussion

The species–area relationship (SAR) is one of the most universal and best documented ecological patterns [62,140], and the Italian dung beetles are no exception. When considering peninsular regions alone, species richness in dung beetles increases with area (thus supporting hypothesis 1). As expected, Sardinia has a strongly impoverished fauna, whereas the Sicilian fauna is only slightly impoverished (hypothesis 2). This is perfectly in line with what was observed for odonates, for which the fauna of Sardinia is highly impoverished, but that of Sicily is not [60,63]. These results can be explained by the different degree of isolation of the two islands: while Sardinia is very isolated from the Italian mainland (with a minimum distance of about 190 km), Sicily is separated from the mainland by about only 3 km. This impoverishment is also in agreement with the low number of dung beetle species recorded in the Iberian islands when compared with mainland areas [141]. If mainland SARs are used to predict the expected number of species, Sardinia should have 60 species of aphodiines, 35 species of scarabaeines, and 104 species of dung beetles in total. These values are much lower than the observed ones (39 species of aphodiines, 19 species of scarabaeines, and 65 species of dung beetles in total). It should be noted that there are many uncertain records from Sardinia [10] that were excluded from the analyses. If these records were included, the Sardinian fauna would include about 53 species of aphodiines, 30 species of scarabaeines, and 94 species of dung beetles in total. Thus, the fauna of Sardinia would appear still impoverished, although to a lesser degree. Further research in Sardinia is therefore needed to ascertain the occurrence of species doubtfully recorded from this region. Slopes (*z*-values) of the mainland SARs for the Italian dung beetles were very low (ranging between 0.029 and 0.098), if compared with those commonly observed in other systems (usually between 0.2 and 0.4) [142], but relatively similar to those observed for dung beetles in the Iberian peninsula (0.089–0.142) [141], possibly a reflection of the good dispersal capability and low ecological specialization of these insects, which are usually able to use dung resources almost everywhere [1,141].

A comparison of *c*-values indicates that there are much more aphodiine species than scarabaeine species per unit area, which may reflect larger population sizes for aphodiines [116], in addition to the obvious reason that, in the study area, the aphodiine fauna is richer than the scarabaeine fauna. Similarly, SARs constructed for Iberian dung beetles support that the *c*-value is higher in aphodiines when compared with scarabaeines [141]. It would be useful in the future to extensively compare SARs for aphodiines and scarabaeines at smaller scales and in faunas where there is no predominance of aphodiines to better understand how differences in the ecology of these two groups influence their SAR.

We found a negative relationship between scarabaeine richness and latitude, which supports previous findings of a negative relationship between latitude and dung beetles in general on local communities [143]. These results indicate that scarabaeines conform to the latitudinal gradient (thus supporting hypothesis 4), instead of being influenced by the peninsula effect (being therefore in contrast with hypothesis 3). Thus, scarabaeines behave similarly to darkling beetles [56] but differently from other groups, such as birds, small mammals, odonates, ground beetles, hydradephagan beetles, and ants, which show a peninsula effect [60,65,143]. However, we found no clear latitudinal trend in species richness for both the total dung beetle fauna and the aphodiines, which contrasts with both the peninsula effect (hypothesis 3) and the latitudinal gradient (hypothesis 4). This lack of relationship for dung beetles as a whole is driven by aphodiines, which dominate the Italian dung beetle fauna. Interestingly, aphodiine richness seems to vary with latitude according to a U-shaped pattern, which suggests that this fauna might include two biogeographical distinct components: one represented by more northern species, which decline southward (following the peninsula effect), and one represented by more southern species, which decline northward (following the latitudinal gradient). This hypothesis might be explored in the future by modeling individual species ranges and considering possible differences in the use of food among species. Overall, these results suggest that aphodiines were possibly subject to large post-glacial movements which partially erased the historical signal retained by scarabaeines because of aphodiine wider ecological (especially thermal) needs.

Climate influences dung beetle richness patterns in various ways. As expected, precipitation had a positive influence on both aphodiine richness and total dung beetle richness, but not on scarabaeines (hypothesis 5). Although rain may have per se a negative effect on dung beetle ecology by removing nutrients from the dung, total precipitation reflects, at this scale, the amount of water available and hence humidity. Thus, this result is consistent with the fact that aphodiines (which are mostly dwellers and represent the vast majority of dung beetle diversity in the study area) are favored by humid climates, where excrement degradation proceeds slowly, whereas scarabaeines (which are rollers and tunnellers) can also cope with arid climates [1,83,85,86]. This interpretation is also confirmed by the influence of aridity, a fundamental aspect of the Mediterranean climate. Aridity influenced negatively aphodiine richness and total dung beetle richness, but not scarabaeine richness (thus supporting hypothesis 5). Temperature exerted a negative influence on aphodiines but not on scarabaeine richness, leading to a lack of response for the total dung beetle richness (hypothesis 5). The contrasting responses of aphodiines and scarabaeines is consistent with their preferences for cold (temperate) and hot (tropical and Mediterranean) climates, respectively [85,86].

Climatic variability influences negatively both the total dung beetle richness, and the two main groups (aphodiines and scarabaeines). While this negative influence on total and aphodiine richness supported our hypothesis 5, the effect on scarabaeines was unexpected and indicated that their ability to protect the offspring through subterranean pedotrophic nests do not make them immune to the negative effects of widely oscillating temperatures.

We found that elevation acted positively on aphodiines, thus contrasting with hypothesis 6. Previous research showed that the relationship between dung beetle diversity and elevation may show a decreasing pattern (e.g., [85,91,93,94,96,144,145]), a mid-elevation peak [98], or a lack of relationship [99]. Also, previous research showed that elevational range was not an important predictor of dung beetle richness compared to climatic factors [141,146,147]. This may be the consequence of both the heterogeneity in the environmental adaptations of dung beetles and the overwhelming importance of climatic factors that vary with elevation [148,149,150], making elevational range only a less efficient surrogate of climate. Our results suggest that the average elevation of a given region has a positive influence on aphodiine diversity, likely reflecting their ability to cope with very cold climates (some species are found at very high elevations, even on the snow [10]), and hence to exploit a higher habitat diversity.

Faunal relationships between regions were distinctly related with inter-regional distances, whereas differences in environmental setting (climate and topography) had negligeable effects, thus supporting the importance of geographical proximity instead of ecological similarity (random processes in hypothesis 7). In fact, it is possible that the dominance of geographical proximity on current patterns might at least partially reflect human activities, since agricultural practices and the presence of livestock through millennia may have profoundly influenced dung beetle assemblages by favoring species movements and faunal homogenization [151]. Inter-regional similarities indicate that Sardinia has a unique dung beetle fauna, well distinct from those of mainland areas. This agrees with previous findings in other groups, such as ground beetles [152], leaf beetles [152], darkling beetles [56], odonates [60], and burnet moths [153]. Earwigs are an exception, as their insular faunas appear to be very similar to those of mainland areas facing the Tyrrhenian Sea [61], suggesting extremely high dispersal capabilities with possibly recent immigrations. Sicily appears also very distinct from the mainland for dung beetles, with strong affinities with Sardinia. This similarity in the faunal composition between Sicily and Sardinia may be explained by the similarity in climatic conditions (these two islands are characterized by high temperatures and low precipitation) and is consistent with results obtained for ground beetles [152], leaf beetles [152], and burnet moths [153], where Sicily clustered with Sardinia. At the same time, the dung beetle fauna of Sicily shows some affinities with those of southern Italian mainland regions, as already observed in darkling beetles [56], earwigs [61], and odonates [60], thus indicating a low level of isolation for this island. Among the mainland regions, biogeographical relationships indicate some major discontinuities for the dung beetle faunas, with important differences between aphodiines and scarabaeines. Aphodiine biogeographical patterns reflect the major orographic features of the study area, showing three main faunal groups, corresponding to the Alps, the northern Apennines, and the southern Apennines. By contrast, scarabaeine biogeographical patterns are characterized by a major discontinuity roughly corresponding to the Po River, which suggests a major role for climatic factors, with northern regions characterized by humid and cold climates, and central–southern regions by warmer and drier climates [154]. These results suggest that orography is important for aphodiine distribution, whereas climate is more important for scarabaeines, which are typically more thermophilic. The presence of these strong discontinuities may explain why biogeographical similarities appear not affected by changes in environmental characteristics after the influence of geographical proximity is removed and (as postulated with hypothesis 7) suggests a role for historical factors represented by the influence of Pleistocene glaciations, which made southern Italian regions an important refugial area [56,60,61].

## 5. Conclusions

The Italian dung beetle fauna shows complex macroecological patterns that reflect the interaction of the current climate, topographic setting, and historical factors. In general, species richness increases with area, as hypothesized according to the species–area relationship (SAR), one of the most universal ecological patterns. However, islands had fewer species than expected according to the SAR, which conforms to the hypothesized impoverishment of island faunas due to their isolation, a phenomenon that appears however much more marked for Sardinia (very isolated) than from Sicily (close to the mainland). We found no support for the hypothesis that dung beetles are affected by the peninsula effect, which contrasts with the decline in species richness from the base (north) to the tip (south) of the Italian peninsula observed in other groups. On the contrary, scarabaeine richness increases southward, thus supporting the latitudinal gradient hypothesis for this group. This gradient reflects the preference of these insects for hot and dry climates and may also represent a consequence of the role of southern regions as Pleistocene refugia. In general, dung beetles respond to climatic and topographical characteristics as hypothesized according to their ecology and behavior, with aphodiine richness being positively influenced by humid and cold climates (being therefore positively influenced by the relief). In general, spatial turnover in species composition appeared to be influenced by geographical proximity among regions more than by their environmental similarities, which supports the importance of random dispersal processes. However, distinct biogeographical discontinuities between the Alpine regions and different sectors of the Apennines also indicate a role for historical factors (viz. the influence of Pleistocene glaciations).

## Figures and Tables

**Table 1:** Species–area relationships for the Italian dung beetles. *S* = number of species; *A* = area (10^3^ km^2^).

Group	Equation	*R* ^2^	*p*-Value
**Islands included**			
All dung beetle species	ln(*S*) = 0.004 × ln(*A*) + 4.570	<0.001	0.933
Aphodiinae	ln(*S*) = −0.019 × ln(*A*) + 4.096	0.013	0.638
Scarabaeinae	ln(*S*) = 0.032 × ln(*A*) +3.368	0.299	0.608
**Islands excluded**			
All dung beetle species	ln(*S*) = 0.056 × ln(*A*) + 4.464	0.412	0.004
Aphodiinae	ln(*S*) = 0.029 × ln(*A*) + 4.001	0.143	0.123
Scarabaeinae	ln(*S*) = 0.098 × ln(*A*) + 3.234	0.295	0.020

**Table 2:** Regressions between richness (residuals from species–area relationships) and latitude for the Italian dung beetle fauna.

Group	Equation	*R* ^2^	*p*-Value
All dung beetle species	Richness = −0.592 × Latitude + 25.748	0.103	0.193
Aphodiinae	Richness = −0.035 × Latitude − 1.471	<0.001	0.904
Scarabaeinae	Richness = −0.830 × Latitude + 36.095	0.370	0.007

**Table 3:** Results of multimodel selection for the influence of topographical and climatic variables on dung beetle richness in Italy. SE: standard error.

Group	Parameter	Estimate	SE	*p*-Value
**Total**				
	Intercept	20.535	19.111	0.294
	**Full average**			
	Temperature Annual Range	−0.859	0.668	0.211
	Annual Precipitation	0.010	0.010	0.355
	Precipitation of Driest Quarter	−0.045	0.048	0.356
	Mean Elevation	−0.0002	0.001	0.783
	**Conditional average**			
	Temperature Annual Range	−1.158	0.506	0.031
	Annual Precipitation	0.018	0.007	0.012
	Precipitation of Driest Quarter	−0.085	0.319	0.013
	Mean Elevation	−0.002	0.002	0.289
**Aphodiinae**				
	Intercept	6.576	9.413	0.495
	**Full average**			
	Annual Mean Temperature	−0.139	0.237	0.562
	Annual Precipitation	0.004	0.005	0.468
	Precipitation of Driest Quarter	−0.026	0.030	0.392
	Mean Elevation	0.001	0.002	0.397
	Temperature Annual Range	−0.216	0.336	0.530
	**Conditional average**			
	Annual Mean Temperature	−0.433	0.217	0.059
	Annual Precipitation	0.009	0.004	0.036
	Precipitation of Driest Quarter	−0.049	0.023	0.042
	Mean Elevation	0.003	0.001	0.042
	Temperature Annual Range	−0.598	0.292	0.058
**Scarabaeinae**				
	Intercept	15.639	22.463	0.491
	**Full average**			
	Temperature Annual Range	−0.854	0.445	0.063
	Mean Elevation	−0.001	0.005	0.868
	Annual Mean Temperature	0.488	0.678	0.476
	Annual Precipitation	0.002	0.003	0.517
	**Conditional average**			
	Temperature Annual Range	−0.977	0.326	0.005
	Mean Elevation	−0.001	0.006	0.829
	Annual Mean Temperature	0.936	0.680	0.175
	Annual Precipitation	0.004	0.003	0.140

**Table 4:** Results of Mantel tests and partial Mantel tests of biogeographical distances against environmental and geographical distances for Italian dung beetles.

Matrix Correlation		Biogeographical Distances			
Matrix A × Matrix B	Matrix C (Controlling)	Sørensen		Simpson	
		*r*	*p*-Value	*r*	*p*-Value
**Total dung beetle fauna**					
Environmental distances	-	0.308	0.059	0.411	0.026
Centroids	-	0.572	<0.001	0.712	<0.001
Environmental distances	Centroids	−0.021	0.443	0.017	0.332
Centroids	Environmental distances	0.507	<0.001	0.638	<0.001
**Aphodiinae**					
Environmental distances	-	0.235	0.099	0.395	0.027
Centroids	-	0.613	<0.001	0.724	<0.001
Environmental distances	Centroids	−0.169	0.914	−0.024	0.447
Centroids	Environmental distances	0.598	<0.001	0.666	<0.001
**Scarabaeinae**					
Environmental distances	-	0.474	0.003	0.168	0.097
Centroids	-	0.621	<0.001	0.484	<0.001
Environmental distances	Centroids	0.192	0.0804	−01.45	0.862
Centroids	Environmental distances	0.486	<0.001	0.478	<0.001

## Data Availability

All data are reported in the Supplementary Materials.

## Supplementary Materials

The following supporting information can be downloaded at: https://www.mdpi.com/article/10.3390/insects15010039/s1, Table S1: Dung beetle species distribution in Italy; Table S2: Environmental variables; Tabel S3: Distances between regions (in km, calculated as distances between centroids of latitude and longitude); Figure S1: Values of node strength in cluster analyses for total dung beetle species composition (**A**,**B**), aphodiines (**C**,**D**), and scarabaeines (**E**,**F**) of Italian regions, using Dice–Sørensen (**A**,**C**,**E**) and Simpson (**B**,**D**,**F**) indices of similarity.
