# Supplementary figures and images for: Macroecology of Dung Beetles in Italy
Source: Insects. 2024 Jan 7;15(1):39. doi: 10.3390/insects15010039 (PMC10816216; doi:10.3390/insects15010039)

A

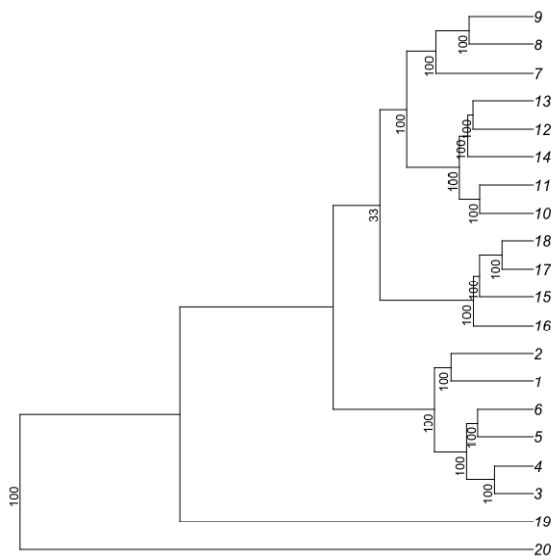

B

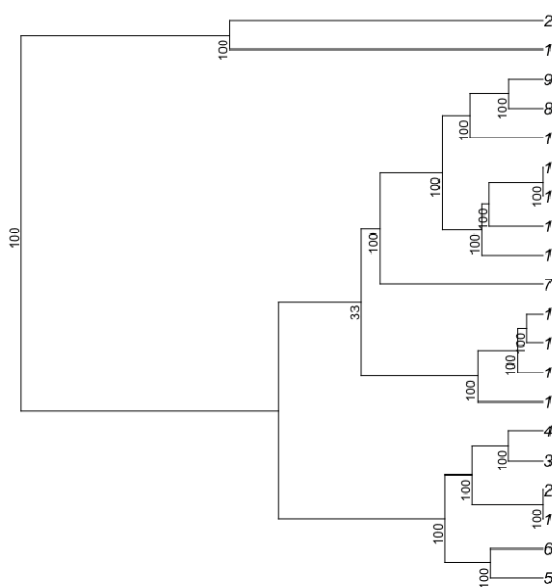

C

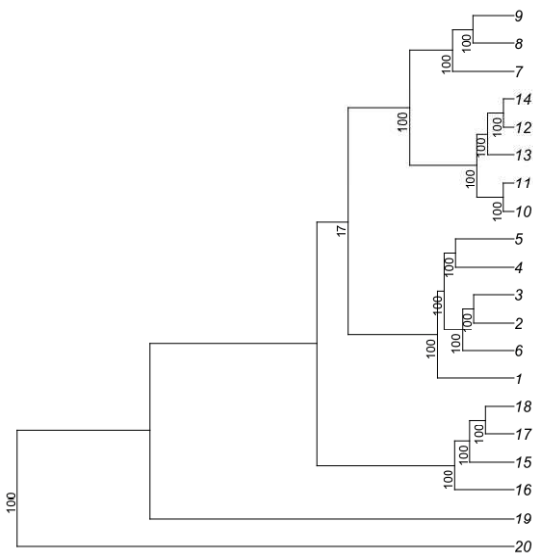

D

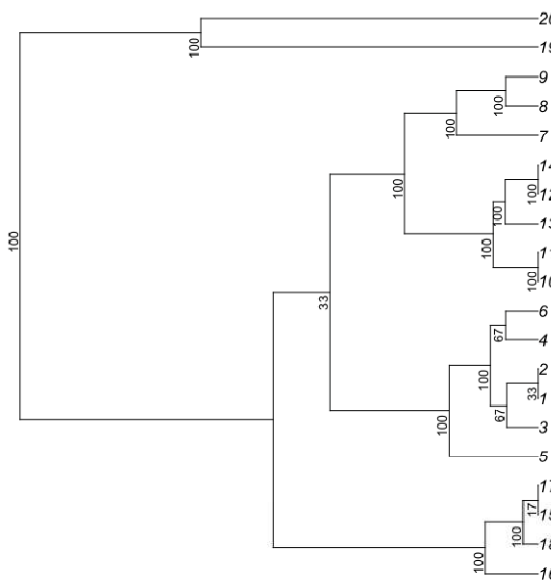

E

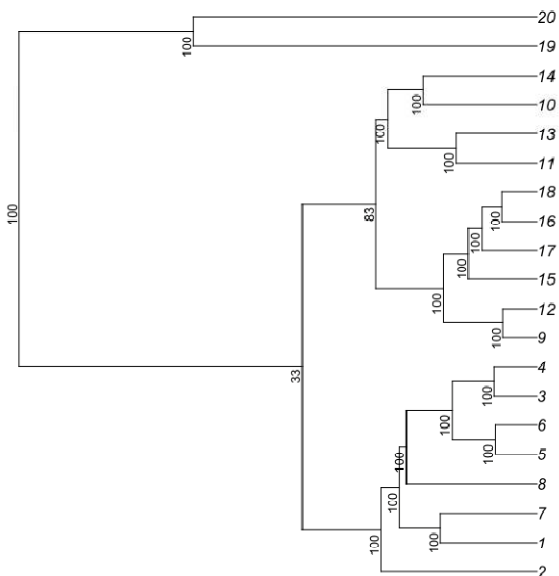

F

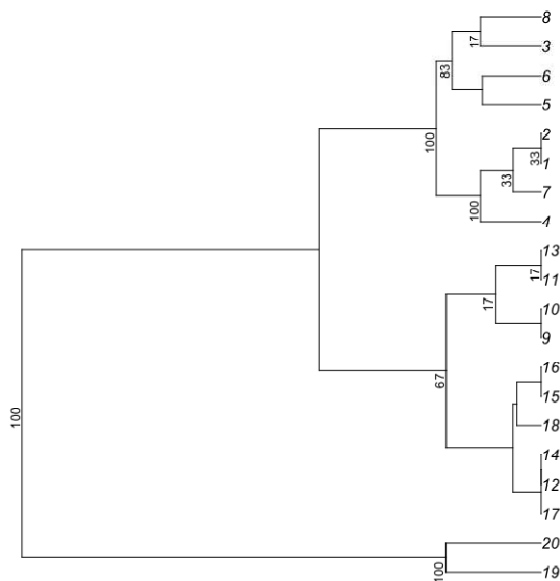

Supplement: Supplementary file 1 [file insects-15-00039-s001.zip › Figure S1.pdf]
